# Incidence of cardiovascular disease up to 13 year after cancer diagnosis: A matched cohort study among 32 757 cancer survivors

**DOI:** 10.1002/cam4.1754

**Published:** 2018-09-15

**Authors:** Dounya Schoormans, Pauline A. J. Vissers, Myrthe P. P. van Herk‐Sukel, Johan Denollet, Susanne S. Pedersen, Susanne O. Dalton, Nina Rottmann, Lonneke van de Poll‐Franse

**Affiliations:** ^1^ CoRPS ‐ Center of Research on Psychology in Somatic diseases Department of Medical and Clinical psychology Tilburg University Tilburg The Netherlands; ^2^ Netherlands Comprehensive Cancer Organisation (IKNL) Utrecht The Netherlands; ^3^ PHARMO Institute for Drug Outcomes Research Utrecht The Netherlands; ^4^ Department of Psychology University of Southern Denmark Odense Denmark; ^5^ Department of Cardiology Odense University Hospital Odense Denmark; ^6^ Survivorship Unit Danish Cancer Society Research Center Copenhagen Denmark; ^7^ National Research Center for Cancer Rehabilitation Department of Public Health University of Southern Denmark Odense Denmark; ^8^ Division of Psychosocial Research and Epidemiology Netherlands Cancer Institute Amsterdam The Netherlands

**Keywords:** cancer survivors, cardiotoxic treatment, cardiovascular disease, matched cohort study

## Abstract

We examined the incidence of cardiovascular disease (CVD) among 32 757 cancer survivors and age‐, gender‐, and geographically matched cancer‐free controls during a follow‐up period of 1‐13 years, and explored whetherCVD incidence differed by received cancer treatment, traditional cardiovascular risk factors, age, or gender. Adult 1‐year cancer survivors without a history ofCVD diagnosed with breast (n = 6762), prostate (n = 4504), non‐Hodgkin (n = 1553), Hodgkin (n = 173), lung and trachea (n = 2661), basal cell carcinoma (BCC; n = 12 476), and colorectal (n = 4628) cancer during 1999‐2011 were selected from the Netherlands Cancer Registry and matched to cancer‐free controls without a history ofCVD. Drug dispenses and hospitalizations from thePHARMO Database Network were used as proxy forCVD. Data were analyzed using Cox regression analyses. Prostate (HR: 1.17; 95%CI: 1.01‐1.35) and lung and trachea (HR: 1.48; 95%CI: 1.10‐1.97) cancer survivors had an increased risk for developingCVD compared to cancer‐free controls. This increased risk among lung and trachea cancer survivors remained statistically significant after including traditional cardiovascular risk factors and cancer treatment information (HR: 1.41; 95%CI: 1.06‐1.89). Among prostate cancer survivors, the increased risk of incidentCVD was limited to those who received hormones and those without traditional cardiovascular risk factors. Breast, non‐Hodgkin,BCC, and colorectal cancer survivors showed no increasedCVD risk compared to cancer‐free controls. There was an increased risk of incidentCVD among prostate, and lung and trachea cancer survivors compared to age‐, gender‐ and geographically matched cancer‐free controls. Studies including longer follow‐up periods are warranted to examine whether cancer survivors are at increased risk of long‐term incidentCVD.

## 1 BACKGROUND

Nowadays, more than 80% of cancer patients become long‐term cancer survivors.^1^ Therefore, the effects of treatment and long‐term related complications are becoming increasingly important representing new challenges to health‐care providers.^2^ It is estimated that cardiovascular disease (CVD) is the number one comorbidity in cancer survivors and responsible for many noncancer‐related mortalities among cancer survivors.^3^ Cancer survivors can develop a range of CVDs, such as arrhythmias, myopericarditis, myocardial infarction, cardiomyopathy, cardiac failure, valvular disease, and congestive heart failure after cancer treatment.^4^, ^5^ Besides similarities in risk factors for both cancer and CVD, the cardiotoxic effect of certain cancer therapies poses a risk to cancer survivors.^6^ Several pathophysiological mechanisms such as oxidative stress as a result of anthracycline generated free radicals and increased inflammation have been suggested to be involved in the pathogenesis of CVD in cancer survivors.^4^, ^7^

Current studies on incident CVD in cancer survivors mostly include just one malignancy, hence large population‐based studies focusing on patients with various cancer types are scarce. Simultaneously, investigating the incidence of CVD among different cohorts of cancer survivors enables direct comparison of CVD risk and potentially sheds a light on generic degenerative pathways that are malignancy “overarching”. Moreover, definitions of CVD differ across studies being based on drug use, registries, self‐report, or hospitalization records, which complicates comparisons between studies and thus cancer types. Furthermore, knowledge of which cancer survivor populations have an increased risk of incident CVD compared to matched cancer‐free controls is largely lacking. This makes it difficult to differentiate whether observed CVD among cancer survivors is due to a general aging effect or due to cancer and its treatment. This information is particularly valuable for future research and survivorship care in order to improve the long‐term outcomes of high‐risk populations.

Armenian and colleagues recently published one of the largest studies examining the magnitude of CVD risk among 36 232 cancer patients diagnosed 2‐8 years ago compared to the CVD‐risk in 73 545 age‐ and gender‐matched cancer‐free controls.^8^ After adjustment for traditional cardiovascular risk factors (ie, hypertension, hypercholesterolemia, and diabetes mellitus), patients with multiple myeloma, lung cancer, non‐Hodgkin lymphoma, ovarian, kidney, and breast cancer had an increased risk of CVD compared to their matched cancer‐free controls.^8^ The researchers argued that this increased risk is likely the result of cardiotoxic cancer treatment, yet information on predictors of CVD risk within particular cancer‐treatment is lacking. Furthermore, in their analyses the authors controlled for traditional CVD risk factors developed after cancer diagnosis. As cardiotoxic treatment is known to increase the risk of developing traditional cardiovascular risk factors such as hypertension, which can then be seen as a precursor of cancer‐treatment induced CVD itself, the authors may be overcorrecting.

The objective of this study was to examine the risk of incident CVD in 1‐year survivors of the seven most prevalent cancer malignancies (ie, breast, prostate, non‐Hodgkin and Hodgkin, lung and trachea, skin (basal cell carcinoma [BCC]), and colorectal cancer)^9^ compared to the risk of their age‐, gender, and geographically matched cancer‐free controls. We corrected for cancer treatment and traditional cardiovascular risk factors present in the year prior to date of cancer diagnosis or corresponding date for the cancer‐free matched controls, thereby avoiding correction of the possibly increasing prevalence of traditional cardiovascular risk factors due to cancer‐treatment. In addition, we explored whether the risk of developing CVD for cancer survivors compared to that of matched cancer‐free controls differed by received cancer treatment, traditional cardiovascular risk factors present prior to cancer diagnosis, age, or gender. We hypothesized that the incidence of CVD was higher in cancer survivors compared to that of cancer‐free controls in those who received chemotherapy, radiation or hormone treatment.

## 2 METHODS

### 2.1 Cancer survivors and cancer‐free matched controls

For this observational matched cohort study, data was extracted from the Southern Region of the Netherlands Cancer Registry (NCR). The NCR includes all newly diagnosed cancer patients and registers cancer diagnosis (type of malignancy and date of diagnosis), stage, and primary cancer treatment.^10^ The NCR is maintained by the Netherlands Comprehensive Cancer Organization, and the Southern Region covers an area of 2.4 million inhabitants, 10 hospitals, six pathology departments, and two radiotherapy institutions.^10^ For this study, data from the PHARMO Database Network was linked to data from the NCR for cancer patients diagnosed between 1998 and 2011.^11^ The PHARMO Database Network is a population‐based network of electronic healthcare databases and combines data from different primary and secondary healthcare settings in the Netherlands. In this study, the Outpatient Pharmacy Database comprising GP or specialist prescribed healthcare products dispensed by the outpatient pharmacy was used. Drug dispensings are coded according to the International Anatomical Therapeutic Chemical (ATC) Classification developed by the World Health Organization.^12^ Additionally, we used discharge information on hospitalizations, which are coded according to the WHO International Classification of Diseases version 9 (ICD‐9). Information on drug dispenses and hospitalizations were used as a combined proxy for CVD in this study, detailed information on the definition of CVD can be found in the method section. Information on survival status and date of death were derived from the municipal Personal Records database.

All adult patients who were diagnosed between 1999 and 2011 with one of the seven most common incident malignancies (breast, prostate, non‐Hodgkin and Hodgkin, lung and trachea, skin (basal cell carcinoma [BCC]), and colorectal cancer)^9^ as a primary cancer diagnosis, and who were 18 years or older were selected from the NCR. We included skin cancer patients with BCC, as this is a malignant form of skin cancer that rarely metastasizes and that is treated solely with surgical removal of the affected skin. Hence, these cancer survivors functioned as a cardiotoxic treatment‐free cancer survivor cohort. For each cancer survivor, one control who was cancer‐free during the study period was matched on age (±1 year), gender, and geographical location (similar zip code) from the PHARMO Database Network.

Follow‐up for a diagnosis of CVD started 12 months after the cancer diagnosis or the corresponding date for cancer‐free controls (which was set as the index date), as primary treatment is generally finished within the first year. Follow‐up time was measured from index date until CVD, death, loss to follow‐up, or until the end of the study period on 31 December 2011, whichever occurred first. In case, the follow‐up of cancer‐free controls was longer than that of their matched case, the duration of the follow‐up period was set equal to the follow‐up of the matched cancer survivor. This observational study used anonymous patient information. Usage of these data does not fall under the Medical Research Involving Human Subjects Act in the Netherlands; therefore, this study was exempted from medical ethics review and no informed consent was required.

#### 2.1.1 Subsample selection procedure

The patient selection was done in three steps and could only be done after the initial study population was matched as hospitalizations and drug dispensing data from the PHARMO Database Network were used as a proxy for CVD status (see the definition of CVD in next section). First, we selected individuals who had no history of CVD in the 12 months prior or after the date of cancer diagnosis for cancer survivors or the corresponding date for cancer‐free controls as we were interested in incident CVD. Second, in order to avoid CVD detection due to increased clinical checkups and reversible CVD during cancer treatment the index date was set at 1 year after cancer diagnosis or corresponding date for controls. We thus excluded those individuals who died or were lost to follow‐up before the index date. Third, matching was checked as each cancer survivor should have one cancer‐free control and vice versa. If there was no match, the survivor/control was excluded.

### 2.2 Measurements

#### 2.2.1 Cardiovascular disease

CVD was defined as at least two drug dispenses of cardiac therapeutics (ie, ATC code C01) at unique dates within 6 months or hospitalization for CVD (ICD‐9 codes 410‐414 and 420‐429, Table S1), whichever occurred first. Participants who dispensed two C01 drugs within a 2‐week period (<15 days in between) were labeled as having CVD when they had at least three C01 dispenses at unique dates. Note that the date of incident CVD diagnosis was set at the date a participant met the criteria, in a participant who was labeled as having CVD based on two drug dispenses, the date of the second dispense was set as the date of incident CVD diagnosis. To avoid false classifications of CVD, solely usage of cardiac‐related drugs such as diuretics (C03) or beta‐blockers (C07) were not enough to be classified as having CVD, as these drugs have a broad treatment range that include noncardiac indications.

#### 2.2.2 Traditional risk factors for cardiovascular disease

Information on traditional cardiovascular risk factors was obtained based on prescription drugs for hypertension (ATC = C02, C03 [except C03c], C07, C08, C09 [except C09x]), hypercholesterolemia (ATC = C10), and diabetes mellitus (ATC = A10), as these are risk factors for incident CVD.^13^ Having one of the traditional cardiovascular risk factors (yes/no) was defined as one or more drug dispensings in the 12 months prior to the date of cancer diagnosis or corresponding date for cancer‐free controls.

#### 2.2.3 Demographics and clinical information

Demographics (age and gender) of 1‐year cancer survivors and matched cancer‐free controls were extracted from the PHARMO Database Network. Clinical information on tumor stage and cancer treatment information (ie, having received chemo‐, radiation‐, or hormone therapy [yes/no]) was obtained from the NCR.

### 2.3 Statistical analyses

Differences in demographics and traditional cardiovascular risk factors between 1‐year cancer survivors and their age‐, gender‐, and geographically matched cancer‐free control were analysed using ANOVA, the chi‐square test or Student's*t* test for independent samples where appropriate.

Kaplan Meier (KM) curves were used to compare the incidence of CVD in cancer survivors to that of matched cancer‐free controls. Multivariable Cox proportional hazard regression analysis was used to compare incidence of CVD among cancer survivors and their matched cancer‐free control. We controlled for age and gender (demographic adjusted), and then additionally included the traditional cardiovascular risk factors hypertension, hypercholesterolemia, and diabetes mellitus present in the 12 months prior to date of cancer diagnosis or corresponding date for the cancer‐free controls (partially adjusted).^13^ Furthermore, we examined whether cancer survivors had an increased CVD risk compared to matched cancer‐free controls, while additionally adjusting for cancer‐treatment (fully adjusted, ie, chemotherapy [yes vs no], radiation [yes vs no], or hormonal treatment [yes vs no]). To enable this analysis, all cancer‐free controls were provided with artificial treatment information similar to their matched cancer survivor. In sensitivity analyses, traditional cardiovascular risk factors measured from index date to end of follow‐up were entered as time‐varying covariates. In our study, 75%‐85% of all cancer survivors and cancer‐free controls already had traditional cardiovascular risk factors in the 12 months prior to the cancer diagnosis date or the corresponding date for cancer‐free controls; hence, only a small portion developed traditional cardiovascular risk factors during the follow‐up period.

Because cancer‐treatment, traditional cardiovascular risk factors, age, and gender are related to developing CVD,^4^, ^5^, ^13^ we examined whether the risk of incident CVD in cancer survivors compared to that of matched cancer‐free controls differed by chemotherapy (yes vs no), radiation (yes vs no) or hormonal treatment (yes vs no), traditional cardiovascular risk factors (1 or more vs none), age (≤65 vs >65 years), and gender by adding interaction terms to the partially adjusted models testing for effect modification. All cancer‐free controls have received artificial treatment information similar to that of their matched control, enabling modification analyses. Hence, if a cancer survivor received chemotherapy (0 = no vs 1 = yes), the matching cancer‐free control received the matching artificial number 1, indicative of having received chemotherapy. All modification effects were analysed separately for each of the seven malignancies. All statistical tests were two‐sided with alpha set at 5% and performed in SPSS 22.0 (IBM SPSS statistics, Chicago, IL, USA).

## 3 RESULTS

### 3.1 Demographics, traditional cardiovascular risk factors, and clinical information

**Figure 1:**
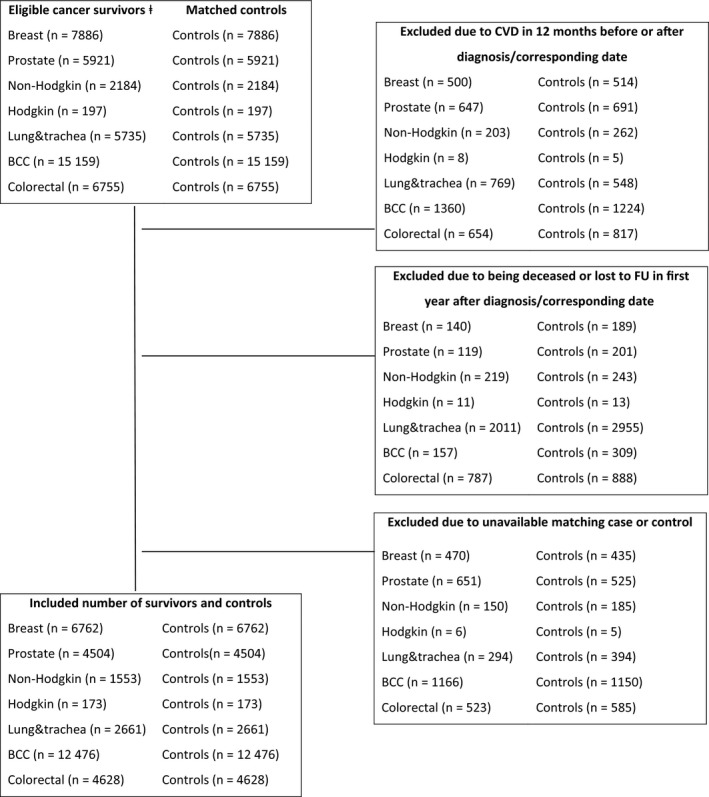
Flow chart of the sample selection of the seven cohorts of cancer survivors and their age‐, gender and geographically matched cancer‐free controls. Note: ⱡ = Initial study population of all adult patients who were diagnosed between 1999 and 2011 with one of the seven most common incident malignancies (i.e. breast; prostate, non‐Hodgkin; Hodgkin; lung & trachea; BCC = basal cell carcinoma; and colorectal cancer) as a primary cancer diagnosis, who were 18 years or older and had a malignancy with stage 1 or higher were selected from the Southern Region of the Netherlands Cancer Registry. CVD = cardiovascular disease.

The study included 6762 breast cancer survivors; 4504 prostate cancer survivors; 1553 non‐Hodgkin survivors; 173 Hodgkin survivors; 2661 lung and trachea cancer survivors; 12 476 BCC survivors; and 4628 colorectal cancer survivors and 1:1 matched age‐, gender‐, and geographically matched cancer‐free control (Figure 1). The median age of cancer survivors and cancer‐free controls was similar within each malignancy, except for breast, BCC, and colorectal cancer survivors, where there were minor differences (Table 1). Cancer survivors more often used drugs for hypertension, hypercholesterolemia, and diabetes mellitus than their matched cancer‐free controls in the year prior to the cancer diagnosis or corresponding date for cancer‐free controls.

**Table 1:** Demographics, traditional cardiovascular risk factors, and clinical information of 1‐year cancer survivors compared to age‐, gender‐, and geographically matched cancer‐free controls

	Breast cancer		Prostate cancer		Non‐Hodgkin		Hodgkin	
	Survivors (n = 6762)	Controls (n = 6762)	Survivors (n = 4504)	Controls (n = 4504)	Survivors (n = 1553)	Controls (n = 1553)	Survivors (n = 173)	Controls (n = 173)
Demographics								
Gender (m)	NA	NA	NA	NA	893 (58)	893 (58)	97 (56)	97 (56)
Median age in years (range)	59 (22‐99)	60 (23‐100)*	68 (42‐94)	68 (43‐95)	65 (18‐94)	65 (19‐95)	34 (18‐75)	35 (19‐76)
Traditional cardiovascular risk factors^a^	2127 (32)	1774 (26)*	2142 (48)	1786 (40)*	621 (40)	499 (32)*	19 (11)	12 (7)
Hypertension	1818 (27)	1468 (22)*	1757 (39)	1493 (33)*	526 (34)	417 (27)*	15 (9)	9 (5)
Hypercholesterolemia	796 (12)	714 (11)*	1135 (25)	977 (22)*	278 (18)	225 (15)*	6 (4)	4 (2)†
Diabetes mellitus	381 (6)	311 (5)*	358 (8)	348 (8)	118 (8)	92 (6)	3 (2)	2 (1)†
Clinical information								
Tumor stage								
I	3017 (45)	NA	441 (10)	NA	247 (26)	NA	25 (15)	NA
II	2701 (41)	NA	2506 (57)	NA	184 (19)	NA	95 (55)	NA
III	728 (11)	NA	727 (17)	NA	209 (22)	NA	31 (18)	NA
IV	205 (3)	NA	708 (16)	NA	329 (34)	NA	22 (13)	NA
Treatment								
Chemotherapy	2600 (39)	NA	19 (0)	NA	878 (57)	NA	170 (98)	NA
Radiotherapy	4871 (72)	NA	1631 (36)	NA	309 (20)	NA	95 (55)	NA
Hormone therapy	3217 (48)	NA	1732 (39)	NA	1 (0)	NA	NA	NA
Surgery	6445 (95)	NA	1130 (25)	NA	61 (4)	NA	NA	NA

	Lung and trachea cancer		BCC cancer		Colorectal cancer	
	Survivors (n = 2661)	Controls (n = 2661)	Survivors (n = 12 476)	Controls (n = 12 476)	Survivors (n = 4628)	Controls (n = 4628)
Demographics						
Gender (m)	1647 (62)	1647 (62)	5853 (47)	5853 (47)	2497 (54)	2497 (54)
Median age in years (range)	65 (20‐94)	65 (21‐95)	64 (19‐99)	64 (18‐99)*	68 (25‐96)	68 (26‐97)*
Traditional cardiovascular risk factors^a^	1096 (41)	864 (33)*	4530 (36)	3812 (31)*	2018 (44)	1673 (36)*
Hypertension	885 (33)	730 (27)*	3790 (30)	3210 (26)*	1698 (37)	1402 (30)*
Hypercholesterolemia	624 (23)	454 (17)*	2195 (18)	1822 (15)*	954 (21)	806 (17)*
Diabetes mellitus	231 (9)	177 (7)*	739 (6)	740 (6)	424 (9)	344 (7)*
Clinical information						
Tumor stage						
I	543 (27)	NA	NA	NA	1035 (23)	NA
II	193 (10)	NA	NA	NA	1413 (32)	NA
III	682 (34)	NA	NA	NA	1281 (29)	NA
IV	603 (30)	NA	NA	NA	748 (17)	NA
Treatment						
Chemotherapy	1493 (56)	NA	1 (0)	NA	1622 (35)	NA
Radiotherapy	1015 (38)	NA	15 (1)	NA	1336 (29)	NA
Hormone therapy	1 (0)	NA	0 (0)	NA	1 (0)	NA
Surgery	738 (28)	NA	1221 (99)	NA	4297 (93)	NA

BCC, basal cell carcinoma; NA, not applicable.

Characteristics at index date are provided in numbers (percentages) for categorical, whereas age is presented in median years (range).

*P < 0.05.

^†^Statistical testing was not possible due to limited cases per cell (<5).

^a^Traditional cardiovascular risk factors present in the 12 months prior to the cancer diagnosis or corresponding date for the cancer‐free controls.

### 3.2 Risk of cardiovascular disease

Follow‐up time ranged from 0 to 13 years. During follow‐up, 3%‐7% of cancer survivors developed CVD. Lung and trachea (5% vs 3%), BCC (7% vs 6%), and colorectal cancer survivors (7% vs 5%) showed an increased risk of developing CVD compared to their matched cancer‐free controls (Table 2).

**Table 2:** Hazard ratios for CVD among 1‐year cancer survivors compared with age‐, gender‐ and geographically matched cancer‐free controls

	CVD	Follow‐up period in median years	Demographic adjusted HR (95% CI)	Partially adjusted HR (95% CI)	Fully adjusted HR (95% CI)
Breast cancer					
Years 0‐7					
Survivors	358 (7)	3 (0‐7)	0.99 (0.86‐1.15)	0.95 (0.82‐1.10)	0.95 (0.82‐1.10)
Controls	343 (6)	3 (0‐7)			
Years 8‐13					
Survivors	41 (3)	9 (8‐13)	1.43 (0.96‐2.12)	1.38 (0.93‐2.05)	1.38 (0.93‐2.05)
Controls	29 (3)	9 (8‐13)			
Prostate cancer					
Survivors	149 (3)	3 (1‐13)	1.17 (1.01‐1.35)^a^	1.12 (0.96‐1.30)	1.12 (0.96‐1.29)
Controls	187 (4)^a^	2 (0‐13)			
Non‐Hodgkin					
Years 0‐3					
Survivors	79 (8)	1 (0‐3)	1.04 (0.77‐1.41)	1.00 (0.74‐1.35)	1.01 (0.75‐1.38)
Controls	74 (7)	1 (0‐3)			
Years 4‐13					
Survivors	34 (6)	6 (4‐13)	1.50 (0.94‐2.38)	1.46 (0.92‐2.32)	1.46 (0.92‐2.32)
Controls	18 (4)	6 (4‐13)			
Lung and trachea cancer					
Survivors	119 (5)	0 (0‐13)	1.48 (1.10‐1.97)^a^	1.45 (1.07‐1.91)^a^	1.41 (1.06‐1.89)^a^
Controls	75 (3)^a^	0 (0‐13)			
BCC cancer					
Survivors	898 (7)	3 (0‐13)	1.08 (0.98‐1.19)	1.04 (0.95‐1.15)	NA
Controls	758 (6)^a^	3 (0‐13)			
Colorectal cancer					
Survivors	290 (7)	2 (0‐8)	1.18 (1.00‐1.41)	1.13 (0.95‐1.34)	1.13 (0.95‐1.34)
Controls	234 (5)^a^	2 (0‐8)			

BCC, basal cell carcinoma; NA, not applicable.

a *P* < 0.05. Demographic adjusted model: adjusted for demographics (ie, age and gender). Partially adjusted model: adjusted for demographics and traditional cardiovascular risk factors (ie, hypertension, hypercholesterolemia, and diabetes mellitus present in the 12 months prior to cancer diagnosis or corresponding date for the cancer‐free controls). Fully adjusted model: adjusted for demographics, traditional cardiovascular risk factors and cancer treatment (ie, chemotherapy, radiation and additionally for hormonal treatment for breast and prostate cancer).

The KM‐curves presenting time to incident CVD for prostate, and lung and trachea cancer diverged from that of their matched cancer‐free controls (log rank statistic:*P* < 0.05), although this was not the case for breast, Hodgkin, non‐Hodgkin, BCC, and colorectal cancer survivors (Figure S1). Visual inspection of the KM‐curves (Figure S1) allowed confirmation of the Cox proportional hazard assumptions for all malignancies, but breast cancer, Hodgkin, and non‐Hodgkin. Given the small number of CVD cases among Hodgkin survivors (n = 9) and their matched cancer‐free controls (n = 8), analyses with respect to risk of incident CVD of Hodgkin survivors vs matched cancer‐free controls was excluded. For breast cancer and non‐Hodgkin, lines crossed at seven and three years follow‐up, respectively. Therefore, in subsequent analyses, incident CVD risk of cancer survivors to that of cancer‐free controls was calculated for both time‐periods (breast cancer 0‐7 and ≥8 years; and non‐Hodgkin 0‐3 and ≥4 years) separately.

Prostate (HR: 1.17; 95% CI: 1.01‐1.35) and lung and trachea (HR: 1.48; 95% CI: 1.10‐1.97) cancer survivors had an increased risk for developing CVD compared to matched cancer‐free controls in the age and gender adjusted analyses (Table 2). After additionally including the traditional cardiovascular risk factors hypertension, hypercholesterolemia, and diabetes mellitus the increased risk of CVD disappeared for prostate cancer (HR: 1.12; 95% CI: 0.96‐1.30), Table 2. The increased risk of CVD among lung and trachea cancer remained statistically significant (HR: 1.45; 95% CI: 1.07‐1.91), and did not change after the addition of received cancer treatment information in the fully adjusted model (HR: 1.41; 95% CI: 1.06‐1.89). Breast cancer, non‐Hodgkin, BCC, and colorectal cancer survivors were not at risk for incident CVD compared to matched cancer‐free controls in any of the models (all*P* > 0.05). Sensitivity analyses which included the traditional cardiovascular risk factors as time‐varying covariates showed similar results across all malignancies (Table S2).

#### 3.2.1 Cancer treatment and cardiovascular disease

The increased risk of developing CVD for cancer survivors compared to cancer‐free controls did not differ by having received chemotherapy or radiation, as indicated by nonsignificant interaction effects (Table 3). The risk of incident CVD among prostate cancer survivors compared to that of their matched cancer‐free controls did depend on received hormone treatment (significant interaction HR: 1.50; 95% CI: 1.11‐2.04, Table 3). Stratified analyses showed that prostate cancer survivors showed an increased risk of incident CVD compared to their matched cancer‐free controls if they had received hormone treatment (HR: 1.41; 95% CI: 1.12‐1.78) but not if they did not have received hormone treatment (HR: 0.94; 95% CI: 0.77‐1.14).

**Table 3:** Age, gender, traditional cardiovascular risk factor, and cancer treatment interaction‐effects for CVD risk among 1‐year cancer survivors compared to that of age‐, gender‐, and geographically matched cancer‐free controls

	Breast cancer		Prostate cancer	Non‐Hodgkin		Lung and trachea cancer	BCC cancer	Colorectal cancer
	Years 0‐7	Years 8‐13		Years 0‐3	Years 4‐13			
Chemotherapy								
Case (survivors vs controls)	0.93 (0.79‐1.10)	0.93 (0.79‐1.10)	NA	1.24 (0.81‐1.90)	1.24 (0.81‐1.90)	1.39 (0.97‐1.99)	NA	1.10 (0.90‐1.34)
Case * chemotherapy	1.08 (0.79‐1.50)	1.08 (0.79‐1.50)	NA	0.68 (0.39‐1.18)	0.68 (0.39‐1.18)	1.05 (0.57‐1.92)		1.12 (0.75‐1.65)
Radiation								
Case (survivors vs controls)	1.08 (0.82‐1.41)	1.08 (0.82‐1.41)	1.05 (0.85‐1.29)	1.10 (0.79‐1.54)	1.10 (0.79‐1.54)	1.21 (0.85‐1.73)	NA	1.06 (0.87‐1.29)
Case * radiation	0.85 (0.62‐1.16)	0.85 (0.62‐1.16)	1.14 (0.85‐1.53)	0.63 (0.31‐1.28)	0.63 (0.31‐1.28)	1.58 (0.85‐2.93)		1.29 (0.86‐1.93)
Hormone treatment								
Case (survivors vs controls)	0.94 (0.78‐1.14)	0.94 (0.78‐1.14)	0.94 (0.77‐1.14)	NA	NA	NA	NA	NA
Case * hormone treatment	1.03 (0.77‐1.37)	1.03 (0.77‐1.37)	1.50 (1.11‐2.04)*					
Traditional CVD risk factors								
Case (survivors vs controls)	1.05 (0.87‐1.27)	1.05 (0.87‐1.27)	1.39 (1.15‐1.74)*	1.10 (0.73‐1.65)	1.10 (0.73‐1.65)	1.49 (1.01‐2.20)*	1.08 (0.95‐1.23)	1.14 (0.89‐1.47)
Case * risk factor	0.81 (0.61‐1.08)	0.81 (0.61‐1.08)	0.68 (0.50‐0.91)*	0.81 (0.47‐1.41)	0.81 (0.47‐1.41)	0.93 (0.52‐1.67)	0.92 (0.76‐1.12)	0.98 (0.69‐1.39)
Age (≤65 vs >65)								
Case (survivors vs controls)	0.93 (0.77‐1.13)	0.93 (0.77‐1.13)	1.04 (0.81‐1.34)	0.94 (0.62‐1.43)	0.94 (0.62‐1.43)	1.43 (0.95‐2.15)	0.96 (0.84‐1.10)	0.98 (0.74‐1.34)
Case * age	1.04 (0.78‐1.39)	1.04 (0.78‐1.39)	1.12 (0.82‐1.53)	1.12 (0.64‐1.95)	1.12 (0.64‐1.95)	0.99 (0.56‐1.78)	1.17 (0.97‐1.43)	1.21 (0.84‐1.74)
Gender								
Case (survivors vs controls)	NA	NA	NA	0.97 (0.67‐1.40)	1.01 (0.74‐1.38)	1.72 (1.19‐2.48)*	1.04 (0.91‐1.19)	1.09 (0.87‐1.36)
Case * gender				1.09 (0.62‐1.93)	0.96 (0.72‐1.27)	0.59 (0.32‐1.09)	1.00 (0.81‐1.21)	1.09 (0.77‐1.55)

Case (survivors vs controls): the risk of developing CVD for cancer survivors compared to that of cancer‐free controls (reference group). Interaction effects were added to the partially adjusted model (adjusting for age, gender, and traditional cardiovascular risk factors). When testing interaction effects, both standardized main effects and the interaction term were added. When testing the interaction effect of traditional cardiovascular risk factors we used the dichotomized variable (ie, having at least one of the traditional cardiovascular risk factors present during the 12 months prior to cancer diagnosis or the corresponding date for cancer‐free controls, yes/no).

*p < 0.05, NA = not applicable.

#### 3.2.2 Traditional cardiovascular risk factors, demographics and cardiovascular disease

The risk of developing CVD according to the presence of traditional cardiovascular risk factors differed between prostate cancer survivors and controls (significant interaction effect HR: 0.68; 95% CI: 0.50‐0.91, Table 3). Stratified analyses showed that prostate cancer survivors had an increased risk of incident CVD when they did not use medication for traditional cardiovascular risk factors (HR: 1.37; 95% CI: 1.09‐1.72), but showed no increased risk when they did take medications for traditional cardiovascular risk factors (HR: 0.94; 95% CI: 0.77‐1.14). There were no significant interaction effects for age or gender (Table 3).

## 4 DISCUSSION

In this population‐based matched cohort study, we observed an increased risk of incident CVD among prostate, and lung and trachea cancer survivors compared to age‐, gender‐ and geographically matched cancer‐free controls, with the increased risk among lung and trachea cancer survivors remaining statistically significant after controlling for traditional cardiovascular risk factors and received cancer treatment. The increased risk of incident CVD was limited to those prostate cancer survivors who received hormone treatment or had no traditional cardiovascular risk factors. Breast, non‐Hodgkin, BCC, and colorectal cancer survivors showed no increased CVD risk compared to matched cancer‐free controls.

Similar to our results, the recent study by Armenian et al^8^ found an increased CVD risk among lung, and prostate cancer survivors, and no increased risk for colon cancer survivors when compared to matched cancer‐free controls. Contrary to our findings, Armenian et al^8^ did find an increased CVD risk among breast cancer and non‐Hodgkin survivors. This discrepancy may be the result of differences in patient samples between both studies, although Armenian and colleges provided no information on cancer treatment or characteristics per malignancy. Differences in CVD definition can also contribute to this discrepancy, as Armenian et al based CVD incidence on recorded clinical diagnosis and procedures, whereas we used drug dispensings and hospitalizations as a proxy for CVD.

One would expect that the increased risk of CVD in cancer survivors is due to the cardiotoxic cancer treatments, as several chemotherapeutic agents, radiation to the chest and hormone treatments are known to be cardiotoxic.^4^, ^5^ As expected, there was no increased risk of incident CVD among the cardiotoxic treatment‐free cancer survivor cohort of BCC survivors. Our study enabled us to include information on cancer treatment. However, results of these analyses, where we adjusted for cancer treatment, did not alter the results. Additionally, results of cancer treatment effect modification showed that the risk of incident CVD in cancer survivors to that of matched cancer‐free controls was not modified by having received chemo‐ or radiotherapy. Surprisingly, we found no increased risk of incident CVD among breast cancer and non‐Hodgkin survivors, while these patients are often treated with cardiotoxic chemotherapeutic agents, radiation to the chest and hormone therapy.^1^, ^14^, ^15^ Although this finding was unexpected, this may be inherent to the lack of detailed information about type of systemic therapy or radiation dosages given. As not all chemotherapeutic agents are equally cardiotoxic^4^ and cardiotoxicity of radiation is dosage‐dependent.^16^ It is therefore possible that grouping all chemotherapeutic agents and radiation treatments irrespective of type and dosage will result in an underestimation of their true effect. Furthermore, previous studies have shown that the incidence risk of CVD is cumulative over time,^14^ where the majority develops CVD many years after cancer diagnosis.^15^ Hence, the follow‐up period in our study could prevent the detection of long‐term incident CVD. Consistent with previous findings,^17^ we did find a possible cardiotoxic effect of hormone treatment among prostate cancer survivors, as the increased risk of incident CVD was limited to those prostate cancer survivors who received hormone therapy.

Factors other than cardiotoxic treatment may also be involved in the pathogenesis of CVD in cancer survivors. First, some risk factors for CVD are also risk factors for cancer,^18^ such as having diabetes.^19^ Indeed, in this study, cancer survivors more often used diabetic drugs in the year prior to their cancer diagnosis than their matched cancer‐free controls (Table 1). As we used drug dispense information as a proxy for traditional cardiovascular risk factors, this difference may also be due to increased diagnostic work‐ups for cancer survivors prior to their cancer diagnosis. Nevertheless, the increased risk for CVD among lung and trachea cancer survivors remained statistically significant even after adjustment for these traditional cardiovascular risk factors, thus not fully explaining the increased CVD risk. Second, lifestyle (eg, high body mass index (BMI), limited exercise, drinking and smoking)^18^ and pathophysiological mechanisms (eg, inflammation and cell aging)^20^, ^21^, ^22^, ^23^ are involved in the pathogenesis of both CVD and cancer. Given the shared underlying risk factors^18^ for both CVD^13^ and colorectal cancer,^24^ it was surprising that colorectal cancer survivors had no increased risk of incident CVD, although this is in line with previous results.^8^, ^25^ Furthermore, receiving cancer therapy may also be associated with adverse lifestyle changes, such as reduced physical activity,^26^ or may induce inflammation,^27^ which is in turn a risk factor for incident CVD.^18^, ^21^ Unfortunately, we have no information on these factors in our study. Third, research within the field of cardiology has suggested that individuals who experience high levels of stress, or experience symptoms of depression or anxiety have an increased risk of incident CVD.^28^ It is well known that both a diagnosis of cancer and its associated treatment may increase stress, anxiety, and depression,^26^, ^29^, ^30^ which could potentially exacerbate the risk of incident CVD in cancer survivors.^6^, ^31^By correcting for the presence of traditional cardiovascular risk factors prior to cancer diagnosis, and by using a strict definition of CVD, we may be missing the precursors of CVD and thus underestimate the risk of CVD in cancer survivors. Our finding that the increased CVD risk among prostate cancer survivors compared to matched cancer‐free controls was limited to those who did not have any traditional cardiovascular risk factors prior to their cancer diagnosis is interesting. This result may be explained by the fact that physicians may be reserved with respect to treating cancer survivors who are already at risk of CVD prior to cancer treatment with cardiotoxic chemotherapeutic agents and/or radiation to the chest.^32^ Moreover, prostate cancer survivors with one or more traditional cardiovascular risk factors in the year prior to cancer diagnosis were more likely to die during the follow‐up period, thus there was less time to develop CVD, which is called the survivorship effect. Alternatively, the development of CVD could be already in process for those with traditional CVD risk factors in the year prior to cancer diagnosis, whereas among cancer survivors without these risk factors the cardiotoxic treatment may function as a kick off for the development of CVD. This indicates that in addition to the current focus on vulnerable cancer patients (ie, those already at risk of developing CVD) there should be sufficient attention for the a priori less vulnerable cancer patients.

The following study limitations should be taken into account when interpreting the results. First, a general limitation inherent to the observational nature of our study is residual confounding. Information on several factors impacting CVD risk, such as the behavioral factors smoking and BMI, medical family history is unknown. Second, we used drug dispense information and hospitalizations as a proxy for CVD, as we did not have information on outpatient medical diagnoses. Furthermore, we used a rigorous algorithm for CVD related drug dispenses, as we included a minimum number of two C01 drug dispenses within a 6 month period, which may have led to an underestimation of the effects. It is likely that we missed a number of heart failure patients who use cardiac‐related drugs such as ACE‐inhibitors and beta‐blockers but no C01 drug, although only the most severe CVD cases will be included by using information on hospitalizations. In addition, as we were interested in incident CVD, cancer survivors and matched cancer‐free controls with CVD in the 12 months prior or after the date of cancer diagnosis or corresponding date for cancer‐free controls were excluded. Hence, we are looking at a subpopulation of 1‐year cancer survivors and matched cancer‐free controls. One of the major strengths of our study is the inclusion of a large population‐based sample with various malignancies with age‐, gender‐, and geographically matched cancer‐free controls. Moreover, the high quality databases of the NCR and the PHARMO Database Network, which date back to 1998 enabled us to establish a 13‐year long follow‐up period. Additionally, we explored whether CVD risk differed by cancer treatment and presence of cardiovascular risk factors. Furthermore, our index date was set 1 year after cancer diagnosis as the best compromise between not starting too late and missing incident CVD, and avoiding the inclusion of reversible CVD due to ongoing treatment. Moreover, choosing this date allowed us to exclude the effect of detecting CVD due to increased clinical checkups in cancer survivors.

In conclusion, prostate and lung and trachea cancer survivors have an increased risk of incident CVD compared to matched cancer‐free controls, with the increased risk of incident CVD remaining among lung and trachea cancer survivors even after adjusting for initial differences in traditional cardiovascular risk factors (hypertension, hypercholesterolemia, and diabetes mellitus) and cancer treatment. Additionally, there is an increased risk of incident CVD among prostate cancer survivors who received hormone treatment or had no traditional cardiovascular risk factors. No increased risk for incident CVD was found for breast cancer, non‐Hodgkin, and colorectal cancer survivors compared to the risk of matched cancer‐free controls. Future studies including longer follow‐up periods are warranted enabling identification of cancer survivors at increased risk of long‐term incident CVD. Additionally, focus should be on disentanglement of behavioral and pathophysiological mechanisms involved in the pathogenesis of CVD among cancer survivors. In the meantime, it is important to be aware of the risk of (cancer treatment‐induced) CVD at the time of cancer diagnosis prior to determining the appropriate cancer treatment. Targeted increased follow‐up for at risk cancer survivors is crucial for a timely detection and treatment of CVD.

## ETHICS APPROVAL AND CONSENT TO PARTICIPATE

This observational study used anonymous patient information. Usage of these data does not fall under the Medical Research Involving Human Subjects Act in the Netherlands, therefore this study was exempted from medical ethics review and no informed consent was required.

## AVAILABILITY OF DATA AND MATERIAL

The data that support the findings of this study are available from Netherlands Cancer Registry (NCR) and the PHARMO database network but restrictions apply to the availability of these data, which were used under license for the current study, and so are not publicly available. Data are however available from the authors upon reasonable request and with permission of both the NCR and PHARMO.

## CONFLICT OF INTEREST

The authors declare that they have no competing interests.
