# Supplementary material for: Incidence of cardiovascular disease up to 13 year after cancer diagnosis: A matched cohort study among 32 757 cancer survivors
Source: Cancer Med. 2018 Sep 15;7(10):4952–63. doi: 10.1002/cam4.1754 (PMC6198235; doi:10.1002/cam4.1754)

Figure S1: *Kaplan Meijer curves presenting time to incident CVD for cancer survivors versus age-, gender-, and geographically- matched cancer-free controls separately for each malignancy*

Breast cancer (log-rank statistic = 0.83)

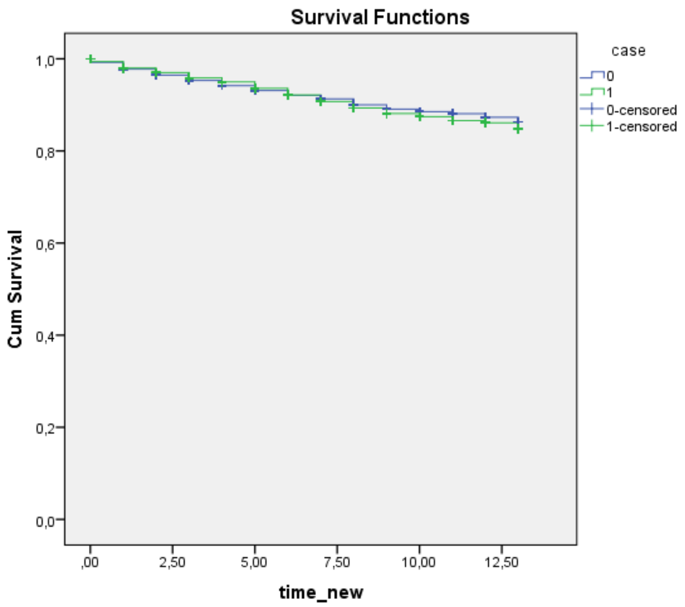

Prostate cancer (log-rank statistic p<0.01)

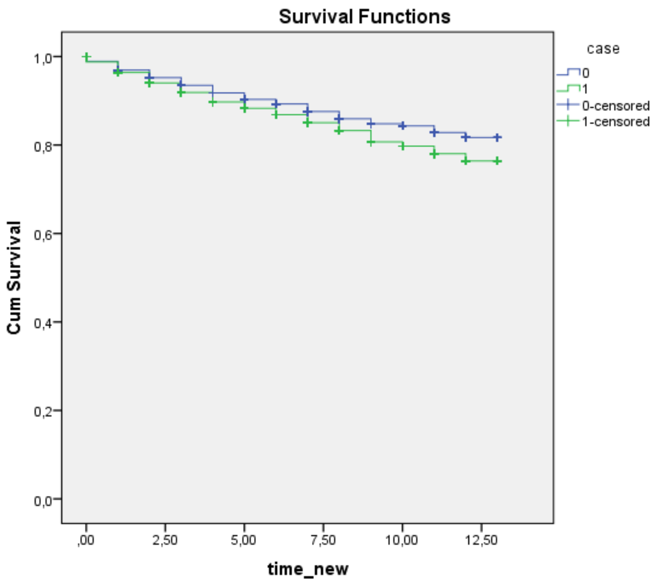

Non-Hodgkin (log-rank statistic p<0.01)

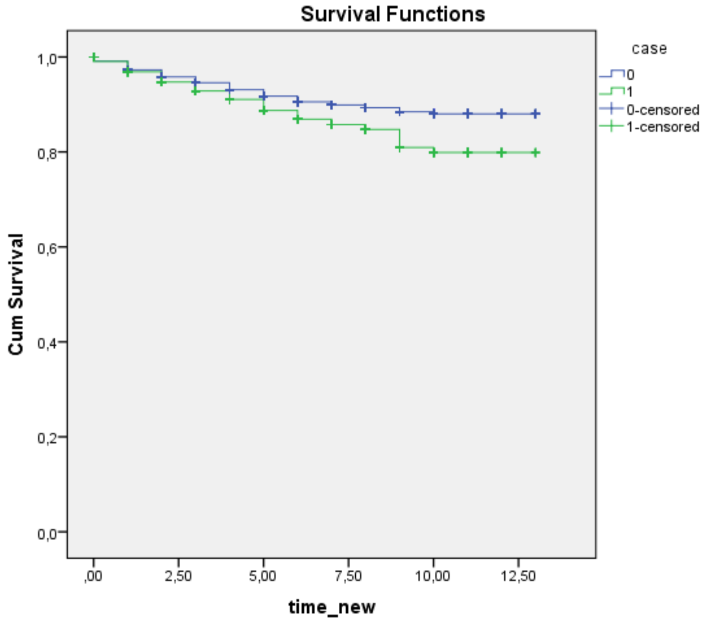

Lung & trachea cancer (log-rank statistic, p<0.01)

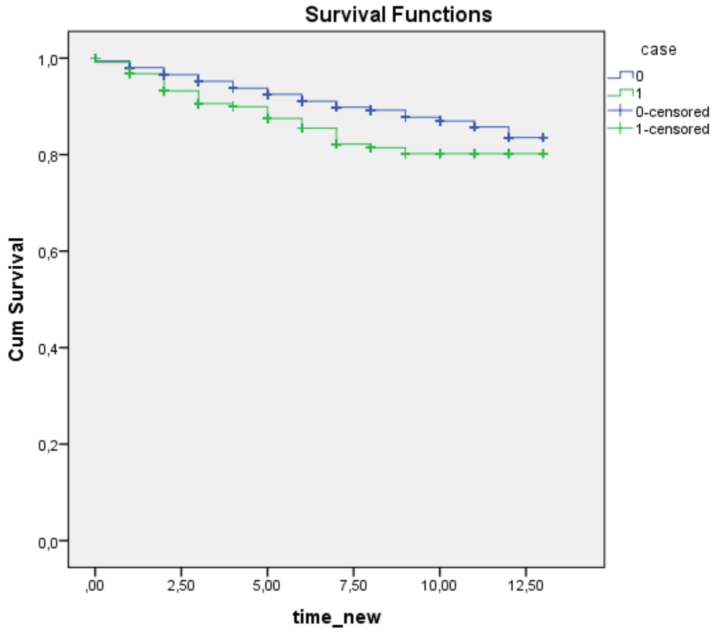

Basal cell carcinoma (log-rank statistics P<0.01)

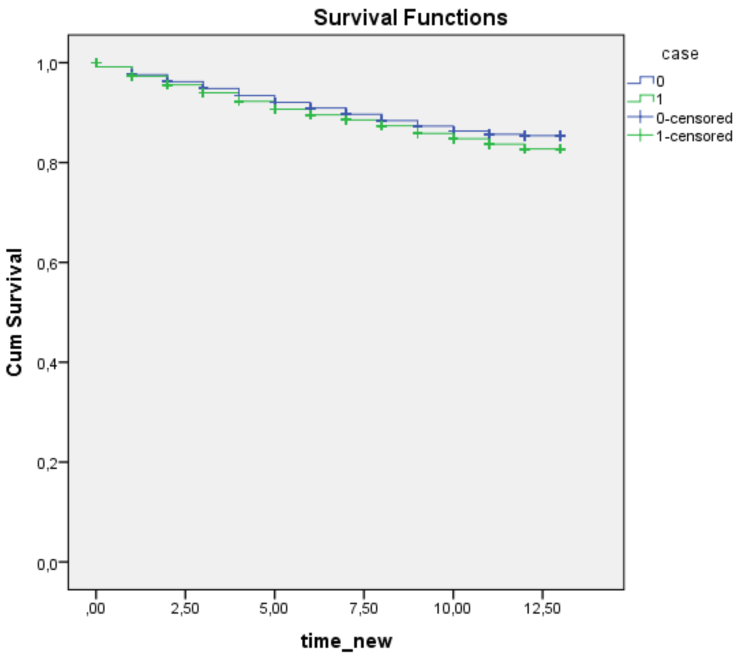

Colorectal cancer (log-rank statistic, p=0.07)

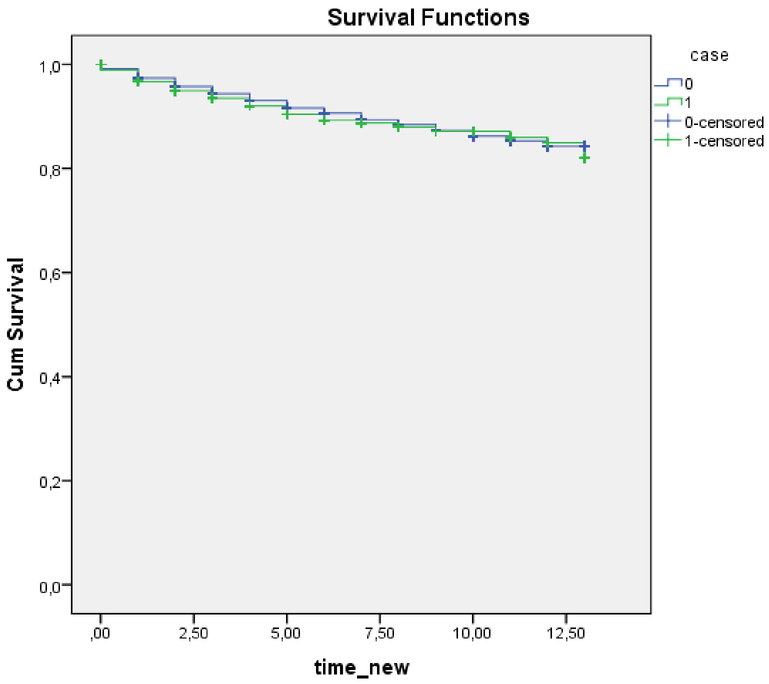

Supplement: Supplementary file 1 [file CAM4-7-4952-s001.pdf]
