# Supplementary material for: Incidence of cardiovascular disease up to 13 year after cancer diagnosis: A matched cohort study among 32 757 cancer survivors
Source: Cancer Med. 2018 Sep 15;7(10):4952–63. doi: 10.1002/cam4.1754 (PMC6198235; doi:10.1002/cam4.1754)
Supplement: Supplementary file 2 [file CAM4-7-4952-s002.docx]

| **ICD-9 code** | **Diagnosis** |
| --- | --- |
| 410-414 | Ischemic heart disease |
| 410 | Acute myocardial infarction |
| 411 | (sub)acute forms of ischemic heart disease |
| 412 | Old myocardial infarction |
| 413 | Angina pectoris |
| 414 | Other forms of chronic ischemic heart disease |
| 420-429 | Other forms of heart disease |
| 420 | Acute pericarditis |
| 421 | (sub)acute endocarditis |
| 422 | Acute myocarditis |
| 423 | Other diseases of pericardium |
| 424 | Other diseases of endocardium |
| 425 | Cardiomyopathy |
| 426 | Conduction disorders |
| 427 | Cardiac dysrhythmias |
| 428 | Heart failure |
| 429 | Ill-defined descriptions and complications of heart disease |

Table S1: *Included hospitalizations for CVD diagnosis based on the ICD-9 codes 410-414 and 420-429*

|  | **Partially adjusted  HR (95% CI)** |
| --- | --- |
| Breast cancer | 0.93(0.84-1.04) |
| Prostate cancer | 1.13(1.01-1.26)* |
| Non-Hodgkin | 1.30(1.06-1.60)* |
| Lung & trachea cancer | 1.57(1.28-1.94)* |
| BCC cancer | 1.05(0.97-1.13) |
| Colorectal cancer† | 1.09(0.96-1.24) |

Table S2: *Sensitivity analyses including traditional cardiovascular risk factors as time-varying co-variants - hazard ratios for CVD among one-year cancer survivors compared with that of age- , gender- and geographically matched cancer-free controls.*

Note: Partially adjusted model: adjusted for demographics and time-varying traditional cardiovascular risk factors (i.e. hypertension, hypercholesterolemia, and diabetes mellitus developed after cancer diagnosis or corresponding date for cancer-free controls). BCC=Basal cell carcinoma †=analyses are limited to the first eight years; *p<0.05.

Figure S1: *Kaplan Meijer curves presenting time to incident CVD for cancer survivors versus age-, gender-, and geographically- matched cancer-free controls separately for each malignancy*
